# Contrast Volume Reduction in Oncologic Body Imaging Using Dual-Energy CT: A Comparison with Single-Energy CT

**DOI:** 10.3390/diagnostics15060707

**Published:** 2025-03-12

**Authors:** Marianna Gulizia, Anais Viry, Mario Jreige, Guillaume Fahrni, Yannick Marro, Gibran Manasseh, Christine Chevallier, Clarisse Dromain, Naik Vietti-Violi

**Affiliations:** 1 Department of Radiology and Interventional Radiology, Lausanne University Hospital, University of Lausanne (UNIL), 1011 Lausanne, Switzerland; marianna.gulizia@chuv.ch (M.G.); anais.viry@chuv.ch (A.V.); yannick.marro@chuv.ch (Y.M.);; 2 Faculty of Biology and Medicine (FBM), University of Lausanne (UNIL), 1015 Lausanne, Switzerland; 3 Department of Nuclear Medicine, Lausanne University Hospital, University of Lausanne (UNIL), 1011 Lausanne, Switzerland; mario.jreige@chuv.ch

**Keywords:** cancer, optimization, scanner, contrast agent, multienergy

## Abstract

**Background/Objectives**: To evaluate the feasibility of reducing contrast volume in oncologic body imaging using dual-energy CT (DECT) by (1) identifying the optimal virtual monochromatic imaging (VMI) reconstruction using DECT and (2) comparing DECT performed with reduced iodinated contrast media (ICM) volume to single-energy CT (SECT) performed with standard ICM volume. **Methods**: In this retrospective study, we quantitatively and qualitatively compared the image quality of 35 thoracoabdominopelvic DECT across 9 different virtual monoenergetic image (VMI) levels (from 40 to 80 keV) using a reduced volume of ICM (0.3 gI/kg of body weight) to determine the optimal keV reconstruction level. Out of these 35 patients, 20 had previously performed SECT with standard ICM volume (0.3 gI/kg of body weight + 9 gI), enabling protocol comparison. The qualitative analysis included overall image quality, noise, and contrast enhancement by two radiologists. Quantitative analysis included contrast enhancement measurements, contrast-to-noise ratio, and signal-to-noise ratio of the liver parenchyma and the portal vein. ANOVA was used to identify the optimal VMI level reconstruction, while *t*-tests and paired *t*-tests were used to compare both protocols. **Results**: VMI_60 keV_ provided the highest overall image quality score. DECT with reduced ICM volume demonstrated higher contrast enhancement and lower noise than SECT with standard ICM volume (*p* < 0.001). No statistical difference was found in the overall image quality between the two protocols (*p* = 0.290). **Conclusions**: VMI_60 keV_ with reduced contrast volume provides higher contrast and lower noise than SECT at a standard contrast volume. DECT using a reduced ICM volume is the technique of choice for oncologic body CT.

## 1. Introduction

Imaging is the cornerstone for cancer diagnosis and treatment response assessment [1,2]. Contrast-enhanced thoracoabdominopelvic (TAP) computed tomography (CT) is the most commonly used imaging modality in oncology because of its speed, wide availability, and ability to cover the entire body for follow-up [3,4]. Given the risk of cancer recurrence years after diagnosis, patients typically undergo repeated CT scans depending on the type and stage of cancer [1]. However, repeated contrast-enhanced CT carries risks to patients. Indeed, iodinated contrast medium (ICM) used for TAP CT is a nephrotoxic agent that can impair renal function in patients with risk factors, such as kidney diseases or diabetes mellitus [5,6,7]. Patients with cancer are more likely to experience acute renal adverse events following CT with ICM than those without cancer [8]. According to the recommendations of the European Society of Urogenital Radiology, there is no consensus on the exact volume of contrast to be injected; however, the lowest volume of contrast medium consistent with a diagnostic result should be used [9]. Additionally, reducing the volume of ICM could benefit the environment by minimizing the impact associated with the production and disposal of iodine-based agents. This is particularly important given the widespread use of ICM in diagnostics, which involves high injection volumes and low biodegradability of the agent, contributing to the contamination of drinking water sources in many regions [10,11].

Dual-energy CT (DECT) is an emerging technique that provides advantages in oncology. By enabling the simultaneous acquisition of high- and low-kilovoltage datasets, DECT produces low virtual monoenergetic images (VMI) that enhance attenuation values, particularly for iodine attenuation [12,13]. This technique has been shown to improve lesion detection by increasing the conspicuity of iodine and enhancing lesion-to-background contrast [14,15,16]. Additionally, DECT enables a reduction in the volume of the ICM compared to single-energy CT (SECT), while maintaining similar image quality. Two oncology imaging studies have demonstrated the potential to reduce ICM volume by 40 to 50% using DECT [17,18]. The first study showed that using VMI_50 keV_ reconstruction with a lean-body-weight-based ICM volume calculation in liver CT provided comparable detection of hepatocellular carcinoma to the standard body-weight-based contrast volume approach [17]. In the second study, Saleh et al. assessed the quality of abdominal DECT using 50% of the weight-based ICM volume compared to SECT using the full recommended volume. This study found that DECT with reduced contrast volume provides an image quality comparable to that of SECT [18]. Both studies had limitations in evaluating specific keV levels: the first study examined only VMI_50 keV_, and the second focused on VMI_60–70 keV_. While it is recognized that radiologists assess multiple VMI levels to optimize contrast and noise, determining the most effective single keV level could streamline processing and interpretation, thereby improving clinical efficiency.

This study aimed to identify the keV level reconstruction that provides the highest overall image quality and to assess the feasibility of reducing ICM volume in oncologic body imaging using DECT with the optimal keV level, in comparison to SECT with a standard ICM volume.

## 2. Materials and Methods

### 2.1. Study Design

This retrospective single-center study was approved by the local ethics committee (CER-VD n°2022-00564), which waived the requirement for informed consent. Eligible patients were identified through our electronic imaging database, which was queried for adult patients who underwent contrast-enhanced TAP DECT for oncology treatment response assessment between February and March 2021. The timeframe for patient recruitment was limited by the introduction of the new DECT protocol in February and the machine upgrade in March. If a prior SECT TAP was performed within one year, it was included in the comparative analysis. The exclusion criterion was a body mass index (BMI) ≥ 30 (kg/m^2^).

### 2.2. Imaging Acquisition

All SECT and DECT examinations were performed using a 256-detector row scanner (Revolution CT; General Electric Healthcare, Milwaukee, WI, USA). DECT was conducted with Gemstone Spectral Imaging, using ultra-fast kV switching between 80 kVp and 140 kVp. The detailed scanning parameters for SECT and DECT are listed in Table 1.

The TAP CT protocol used for oncology imaging at our institution includes only the portal venous phase, acquired 75 s after intravenous contrast injection without bolus tracking. The injection was performed using an automatic power injector (CT Exprès^®^ 3D Contrast Media Delivery System, Bracco, Monroe Township, NJ, USA) with iohexol 300 mg/mL (Accupaque 300, GE Healthcare, Nycomed, Cork, Ireland), injected in an antecubital vein at a rate of 3.0 mL/s. Two different injection protocols were used: (1) SECT with a standard ICM volume [1 mL/kg (0.3 g I/kg) + 30 mL] and (2) DECT with a 30 mL reduction in ICM volume, arbitrarily defined as [1 mL/kg (0.3 g I/kg) of total body weight].

### 2.3. Definition of the Optimal VMI Reconstruction

To perform image quality comparison, DECT was reconstructed at nine different VMI levels ranging from 40 keV to 80 keV at 10 keV intervals. Qualitative and quantitative analyses were performed (1) to identify the VMI level that provides the highest image quality based on the different keV reconstructions in DECT and (2) to compare image quality between SECT with the standard ICM volume and DECT with reduced ICM volume (using the most accurate VMI identified in step 1). All image analyses were conducted using a dedicated workstation (AW Server 3.4; General Electric Healthcare, Chicago, IL, USA).

#### 2.3.1. Qualitative Image Analysis

Two radiologists, each with 9 years of experience in oncology imaging, independently performed a blinded qualitative analysis of global contrast enhancement, noise level, and overall image quality across the five keV level reconstructions (ranging from 40 to 80 keV, at 10 keV intervals). We conducted a comprehensive assessment of the image quality by evaluating image contrast, image noise, and overall image quality across the entire image. This analysis ensured a thorough understanding of imaging performance and diagnostic utility across a wide range of oncologic clinical contexts. A 5-point evaluation scale was used. Contrast enhancement was assessed as follows: 1, very poor; 2, poor; 3, acceptable; 4, good; and 5, very good. Noise was graded as follows: 1, excessive noise; 2, prominent image noise; 3, some image noise; 4, minimal image noise; and 5, limited perceptual image noise. Overall image quality was as follows: (1) no definition between anatomical structures; (2) minimal definition between anatomical structures; (3) definition between anatomical structures; (4) clear definition between anatomical structures; and (5) total definition between anatomical structures. A score of 3 was considered the threshold for diagnostic image quality in all three outcomes. The optimal keV level was determined based on the highest overall image quality score. The inter-reader agreement was assessed.

#### 2.3.2. Quantitative Image Analysis

Quantitative analysis was performed by a radiographer with five years of experience. Contrast enhancement was measured in Hounsfield Units (HUs) across the different keV level reconstructions by placing a 1 cm diameter region of interest (ROI) in the left and right liver lobes, in the portal vein, and in the right erector muscle. Contrast enhancement in the liver parenchyma was expressed as the average HU value of the left and right liver lobes. The standard deviation of the erector muscle was used to define the image noise.

The contrast-to-noise ratio (CNR) and signal-to-noise ratio (SNR) of the liver parenchyma and portal vein were calculated using Equation (1) for the CNR:

(1) $$\mathrm{CNR}=\frac{contrast}{mean\;noise}=\frac{{HU}_{Liver\;Parenchyma}{-HU}_{muscle}}{sd_{muscle}}\;\mathrm{and}\;\frac{{HU}_{Portal\;Vein}\;-{HU}_{muscle}}{sd_{muscle}}$$

And Equation (2) for the SNR was as follows:

(2) $$\mathrm{SNR}=\frac{{HU}_{Liver\;Parenchyma}}{sd_{Liver\;Parenchyma}}\;\mathrm{and}\;\frac{{HU}_{Portal\;Vein}}{sd_{Portal\;Vein}}$$

### 2.4. Comparison of DECT and SECT

Identical qualitative and quantitative image analyses were conducted to compare SECT and DECT at the optimal VMI reconstruction, as determined in the first objective of the study.

### 2.5. Radiation Dose Analysis

The volumetric CT dose index (CTDIvol, mGy), dose-length product (DLP, mGy × cm), and effective dose (mSv) were recorded. The effective dose was estimated using the conversion factor for the adult abdomen at 120 kV (0.0153 mSv.mGy^−1^.cm^−1^) as defined by Deak et al. [19].

### 2.6. Statistical Analysis

Inter-reader reliability was evaluated using Gwet’s agreement coefficient (AC), as it provides more stable agreement estimates, especially when the category prevalence is unbalanced. The level of agreement was categorized as poor (AC < 0.40), fair (0.4 ≤ AC < 0.6), substantial (0.6 ≤ AC ≤ 0.8), and almost perfect (0.8 ≤ AC < 1.00) [19]. Continuous data are presented as the mean ± standard deviation. To define the optimal VMI level, a one-way analysis of variance (ANOVA) was performed to compare the differences in SNR, CNR, and noise in the liver parenchyma between the different VMI reconstructions. Repeated ANOVA was used for the qualitative analysis comparing the rates of contrast enhancement, noise, and overall image quality between the different VMI reconstructions. Due to the small sample size, we used paired *t*-tests to compare image quality between SECT and DECT protocols, as this test is suitable for evaluating two related measurements from the same subjects. All statistical tests were conducted at the two-sided 5% significance level. All analyses were performed using STATA 16.0 software.

## 3. Results

### 3.1. Patient Population

**Figure 1:**
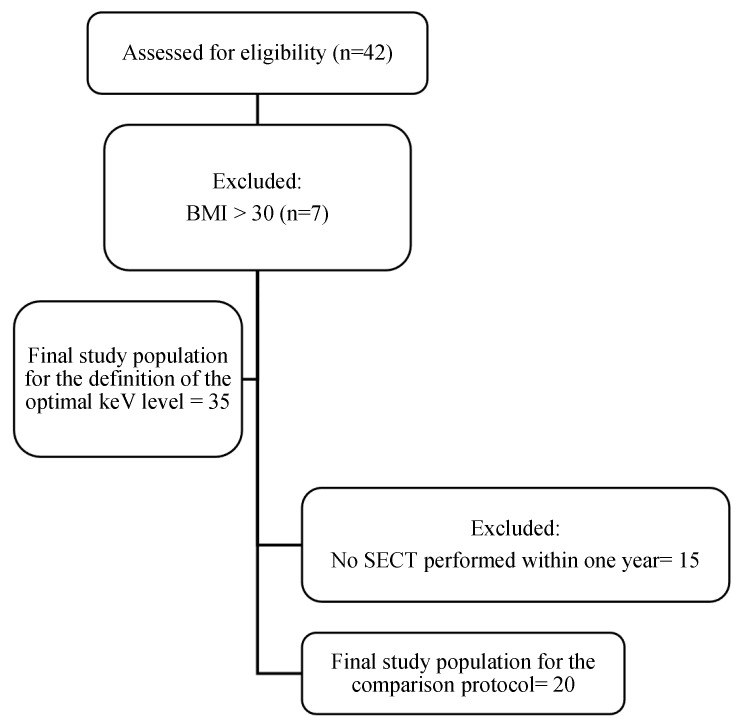
Study flowchart.

The final population included 35 patients [M/F: 21/14, mean age 64.6 ± 9.5 y.o.], with 20 patients having a comparative SECT. The mean time between SECT and DECT examinations was 92 ± 58.5 days. The flowchart of the patient population is shown in Figure 1. The patient population characteristics are described in Table 2.

### 3.2. Optimal VMI Reconstruction Results

#### 3.2.1. VMI Comparison: Qualitative Results

The inter-reader agreement for overall image quality and contrast enhancement was almost perfect (AC = 0.86, AC = 0.94, respectively), while it was substantial for image noise (AC = 0.63). The mean quality scores for all three outcomes (contrast, noise, and overall image quality) were above 3, indicating diagnostic image quality (Table 3). Contrast enhancement was higher in the low-keV images; however, these images also exhibited higher noise levels (Table 3). The highest score for overall image quality was observed for VMI_60 keV_ (Table 3). Therefore, VMI_60 keV_ was selected as the optimal compromise between achieving a higher contrast at lower keV levels and reducing noise at higher keV levels.

#### 3.2.2. VMI Comparison: Quantitative Analysis Results 

Compared to VMI_40 keV_, the contrast enhancement in the liver parenchyma decreased by 56.5%, and the CNR decreased by 22.9% at VMI_80 keV_ (Table 4). No statistically significant difference in contrast enhancement was observed between VMI_60 keV_ and VMI_65 keV_ (all *p* > 0.05). However, a significant difference was found between VMI_60 keV_ and VMI_55 keV_. No statistical differences were observed in CNR_LP_, SNR_LP_, and PV between VMI_55 keV_, VMI_60 keV_, and VM_I65eV_ or between VMI_60 keV_ and VMI_65 keV_ (all *p* > 0.05). SNR and image noise did not show any statistical differences among all VMI (all *p* > 0.05).

**Figure 2:**
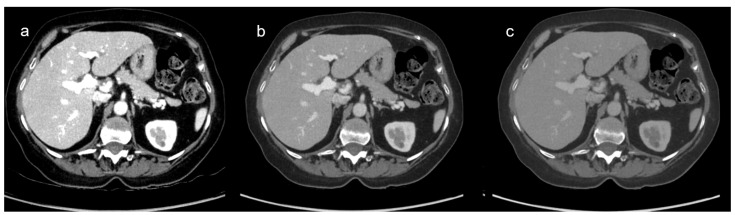
Iodine-attenuation DECT images reconstructed at three different keV levels: (**a**) 80 keV, (**b**) 60 keV, and (**c**) 40 keV. VMI_60 keV_ showed the highest score for overall image quality with a score of 5, while VMI_40 keV_ was rated as 4 and VMI_80 keV_ was rated as 3.

Based on both quantitative and qualitative results, VMI_60 keV_ was identified as the most accurate VMI reconstruction, providing the highest overall image quality and an adequate compromise between contrast and noise (Figure 2).

### 3.3. Comparison of DECT and SECT Results

#### 3.3.1. DECT and SECT Comparison: Qualitative Results

**Figure 3:**
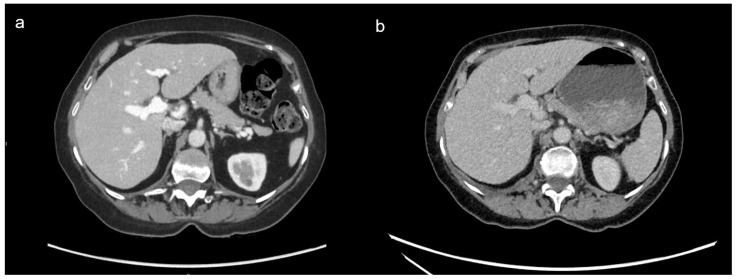
Example of DECT at 60 keV (**a**) and SECT (**b**) axial contrast-enhanced CT images of the same patient during the portal venous phase, presented with identical window width (350 HU) and window level (50 HU). VMI_60 keV_ image (**left**) was rated 5 for both image quality and contrast, whereas the SECT image (**right**) was rated 3 for both characteristics.

Gwet’s agreement coefficients indicated substantial interobserver reliability for overall image quality (AC = 0.769), contrast enhancement (AC = 0.83), and image noise (AC = 0.83) in DECT. In contrast, SECT exhibited lower agreement values (AC = 0.54 for overall image quality, 0.23 for contrast enhancement, and 0.40 for image noise). The overall image quality was not significantly different between DECT with a reduced ICM volume and SECT with a standard ICM volume (*p* = 0.287) (Table 5). Contrast enhancement was higher with DECT than with SECT (*p* < 0.001), whereas image noise was lower with DECT (*p* < 0.001) (Figure 3). The mean score for the three outcomes in both protocols was ≥3, meeting the diagnostic requirements (Table 5).

#### 3.3.2. DECT and SECT Comparison: Quantitative Analysis Results

In terms of contrast volume, the DECT group received a mean contrast volume of 71.4 ± 12.7 mL, while the SECT group received 102.7 ± 14.9 mL (*p* < 0.001), representing a 43.8% reduction in contrast volume with DECT.

Contrast enhancement, CNR, and SNR in the LP and PV were all higher with DECT than with SECT (*p* < 0.001), whereas noise was significantly lower in DECT (*p* < 0.001). Detailed results are presented in Table 6.

### 3.4. Radiation Exposure Results

The mean CTDI, DLP, and effective dose with DECT were 9.7 ± 3.5, 678.1 ± 259.2, and 10.4 ± 4.0, respectively. With SECT, the mean CTDI, DLP, and effective dose were 7.2 ± 1.6, 502.7 ± 116.4, and 7.7 ± 1.8, respectively. The differences between the two protocols were statistically significant (*p* < 0.001).

## 4. Discussion

In this study, we aimed to evaluate the feasibility of reducing the volume of contrast administration in TAP CT for oncologic body imaging using DECT. We compared SECT with a standard ICM volume to DECT with a reduced contrast volume (30 mL reduction per patient) and demonstrated similar image quality, confirming the feasibility of ICM volume reduction with DECT.

Quantitative and qualitative comparisons of the different VMI reconstructions with DECT identified VMI_60 keV_ as the optimal reconstruction. DECT with a reduced ICM volume provided an overall image quality comparable to SECT with a standard ICM volume. Notably, DECT with reduced ICM volume provided higher contrast enhancement and lower noise compared to SECT with standard ICM volume. However, DECT resulted in a 34.72% increase in the radiation dose compared to SECT.

VMI_60 keV_ was identified as the optimal reconstruction level for achieving the highest overall image quality, balancing contrast enhancement and noise reduction, which is consistent with studies conducted by Gao et al. and Lv et al. [20,21]. In contrast, two other studies identified the lowest keV level, typically VMI_40 keV_, as optimal for enhancing lesion depiction, such as liver metastasis or hypervascularization in hepatocellular carcinoma [22], as well as for detection performance in phantom studies [23]. However, as the keV value decreases, the image noise increases, which can negatively affect the overall image quality. Therefore, it is essential to optimize the VMI level to achieve optimal contrast between the liver parenchyma and surrounding tissues while controlling the image noise [24,25]. Our findings align with those of a multi-institutional consensus working to standardize DECT workflows, which recommended VMI_50 keV_ for improved contrast and VMI_70 keV_ for reduced noise in abdominal exams [26]. These results are also supported by Lv et al., who defined an optimal range of 40–70 keV for enhancing the detectability of small HCC without degrading image quality [27]. However, the magnitude of contrast enhancement and thus keV level reconstruction depend on several patient-related factors, including body weight, height, gender, age, and cardiac output [28]. Indeed, to investigate interpatient variability, it is essential to include a larger sample size to enhance the robustness of the present results and perform sub-population analysis. Previous reports evidenced that the predominant patient-related factor was body weight [28,29]. A high BMI can lead to increased image noise and a subsequent reduction in image quality. Consequently, further work will need to determine if this population may necessitate higher keV levels compared with patients with a lower BMI.

While we acknowledge that radiologists often evaluate images at different keV values to optimize contrast or reduce noise, our study focused on determining the most effective single keV level for overall image quality. Additionally, using a single optimal keV level helps streamline the computational processing and interpretation time, which is crucial in clinical settings where efficiency is important. In fact, the environmental impact of radiology often overlooks the role of data storage, which accounts for approximately 2% of the total electricity consumption globally and generates CO_2_ emissions comparable to those of the aviation sector [30]. As data generation continues to grow exponentially in the digital age, adopting a mindset of “digital moderation” is crucial. This involves limiting the production, use, and dissemination of digital technologies whenever possible, ensuring that their benefits outweigh both financial and environmental costs [31].

This study demonstrated the feasibility of DECT performing oncological CT TAP with a 44% reduction in contrast injection volume compared to SECT while maintaining similar interpretability. Our findings are consistent with those of Bae et al., who reported a 40% reduction in contrast volume for liver CT, as well as with Noda et al., who implemented a DECT protocol using 0.3 gI/kg of body weight [17,32]. Additionally, Saleh et al. reported a 50% ICM volume reduction in oncology patients with 0.3 gI/kg of body weight [18]. In our study, this reduction was achievable due to comparable image quality between DECT and SECT, which allowed for clear delineation of anatomical structures as assessed qualitatively and quantitatively. The mean contrast enhancement measured in the liver exceeded 50 HUs in both groups and across all monoenergetic images, in line with the recommendations of Heiken et al. for achieving diagnostic image quality [29]. Moreover, compared to SECT, VMI_60 keV_ exhibited a statistically significant increase in contrast enhancement and reduced noise in qualitative image analysis. Additionally, it showed significantly improved CNR and SNR, along with decreased noise in quantitative analysis. The increased contrast enhancement observed with DECT, which probably leads to higher detectability, has been objectively demonstrated in multiple phantom studies [23,24,33] and clinical studies [22,34]. Further investigations are needed to confirm these assumptions, particularly regarding liver metastases. Nonetheless, according to international guidelines, liver metastases have to be followed using MRI [35].

These results highlight the potential for further ICM volume reduction using DECT, allowing for a lower contrast volume while still achieving sufficient image quality. It is well established that iodinated contrast injections can impair kidney function, potentially leading to contrast-induced acute kidney injury, particularly in patients with preexisting renal impairment, accounting for 11% of acute renal failure cases [36,37]. Reducing the dose of contrast is proposed by international guidelines as a way to prevent acute renal failure emphasizing the interest in further reducing contrast volume injection [8,38].

Our study evidenced a 34.7% increase in radiation doses with DECT compared with SECT. However, the mean CTDI and mean DLP (9.7 mGy, 678.1 mGy cm, respectively) within the DECT group remained below the American diagnostic reference levels (12 mGy, 774 mGy*cm) [39]. Moreover, our radiation doses were approximately 50% lower than those reported in previous studies comparing DECT and SECT [17,40]. In fact, Bae et al. and Nakaura et al. used the SECT technique for the portal venous phase and reported CTDI values of 23.7 mGy and 18.6 mGy, respectively, which are higher than their SECT means CTDI of 7.2 mGy and their DECT CTDI of 9.7 mGy. Thus, a balance must be found between reducing contrast volume and increasing radiation exposure, which may vary depending on the population of interest. In the oncologic population, patients are often elderly with a high prevalence of chronic kidney impairment and limited risk of radiation-induced cancer, shifting the balance in favor of contrast reduction.

This study has several limitations. The first limitation is the small sample size (35 patients), which may introduce heterogeneity due to variations in patient body composition, clinical context, and indications. The sample size was limited owing to the protocol introduction dates, subsequent machine upgrades, and the requirement that patients had previously undergone a CT exam. The small sample size increased the interpatient variability. Thus, a larger cohort study is required to confirm the present results and improve their generalizability. Second, patients with a BMI of >30 were excluded from our cohort, which represents a selection bias. However, as already stated, patients’ BMIs require specific investigation, as the optimal keV level for these patients is probably different from the general population. Another limitation is the comparison between SECT and DECT rather than focusing solely on the volume reduction in DECT. Nonetheless, our results demonstrated statistically comparable image quality between the two protocols.

Further studies are needed to explore the potential of low-keV reconstruction in reducing ICM usage. To leverage the high contrast of lower-keV images, deep learning-based denoising can help reduce noise, which affects image quality, as demonstrated in various phantom studies [41,42]. Another alternative to reduce ICM in body imaging with DECT would be the lean-body-weight strategy for calculating the ICM volume, particularly when using low-keV VMI. A meta-analysis highlighted that the lean-body-weight strategy results in lower volumes than the total-body-weight strategy [43].

## 5. Conclusions

In conclusion, VMI_60 keV_ provided higher contrast enhancement and reduced image noise compared to SECT. DECT allows for a 30 mL reduction in ICM volume per patient compared to SECT while maintaining similar image quality.

## Figures and Tables

**Table 1:** DECT and SECT parameters.

CT Parameters	DECT	SECT
Tube voltage	80–140 kVp	120 kVp
Automatic tube current modulation (mA)	145–515	130–400
Pitch	0.992	1.2
Collimation (mm)	80 × 0.625	80 × 0.625
SFOV (mm)	500	500
Matrix size (pixels)	512 × 512	512 × 512
Gantry rotation time (s/rot)	0.6	0.28
Slice thickness (mm)	2.5	2.5
Slice increment (mm)	2	2
Kernel	Standard	Standard
Reconstruction method	ASIR-V 50%	ASIR-V 50%

Abbreviations: DECT, dual-energy computed tomography; SECT, single-energy computed tomography.

**Table 2:** Patient characteristics.

	Patients (n = 35)
Sex M/F	21/14
Age (years)	64.6 ± 9.5
Body weight (kg)	71.4 ± 12.7
Body Height (cm)	169 ± 9.0
BMI	25.0 ± 3.7
Clinical indications	16/35 Lung cancers 9/35 Urological cancers 6/35 Gynecological cancers 4/35 Digestive cancers 3/35 Skin cancers 2/35 Hematological cancers 1/35 Brain cancer 1/35 Breast cancer

Note: data are presented as the mean ± standard deviation. Abbreviations: BMI, body mass index.

**Table 3:** Qualitative assessment of all VMI reconstructions.

keV	Image Overall Quality (Range)	Contrast Enhancement (Range)	Image Noise (Range)
40	3.22 (3–5)	4.91 (4–5)	3.24 (3–5)
45	3.37 (3–5)	4.87 (4–5)	3.23 (3–5)
50	3.87 (3–5)	4.57 (4–5)	3.44 (3–5)
55	4.36 (3–5)	4.31 (3–5)	3.76 (3–5)
60	4.61 (3–5)	4.06 (3–5)	4.00 (3–5)
65	4.51 (3–5)	3.86 (3–5)	4.40 (3–5)
70	3.99 (2–5)	3.51 (2–5)	4.76 (4–5)
75	3.41 (2–5)	3.10 (2–5)	4.96 (4–5)
80 Gwet’s AC	3.03 (2–4) AC = 0.864	2.94 (2–4) AC = 0.94	4.96 (4–5) AC = 0.63

Note: data are presented as means and ranges.

**Table 4:** Quantitative assessment of VMI reconstructions.

	HU_LP_	CNR_LP_	SNR_LP_	HU_PV_	CNR_PV_	SNR_PV_	Image Noise
40	199.74 ± 24.67	6.90 ± 2.18	11.49 ± 2.04	379.13 ± 29.15	17.59 ± 5.36	13.53 ± 3.61	17.21 ± 4.01
45	174.01 ± 17.98	6.79 ± 1.96	11.80 ± 1.90	312.89 ± 24.29	16.40 ± 4.70	13.36 ± 3.44	14.75 ± 3.35
50	150.86 ± 14.84	6.53 ± 1.83	11.95 ± 1.94	260.55 ± 43.08	15.28 ± 4.33	13.22 ± 3.23	12.76 ± 2.89
55	133.15 ± 12.62	6.34 ± 1.70	12.06 ± 1.86	220.24 ± 34.94	14.27 ± 3.96	13.04 ± 3.01	11.14 ± 2.42
60	119.30 ± 11.08	6.09 ± 1.65	12.27 ± 1.92	190.85 ± 33.73	13.38 ± 4.14	13.01 ± 3.21	9.96 ± 2.17
65	108.1 ± 10.01	5.75 ± 1.57	12.35 ± 1.93	163.33 ± 23.61	11.86 ± 3.40	12.67 ± 2.70	9.15 ± 2.01
70	99.50 ± 9.20	5.55 ± 1.49	12.61 ± 1.94	143.58 ± 19.63	10.87 ± 3.06	12.40 ± 2.46	8.33 ± 1.78
75	92.42 ± 8.76	5.32 ± 1.46	12.29 ± 2.69	127.69 ± 16.70	9.90 ± 2.87	12.20 ± 2.31	7.73 ± 1.67
80	86.93 ± 8.49	5.17 ± 1.44	12.80 ± 2.05	114.78 ± 14.19	9.02 ± 2.66	11.96 ± 2.18	7.22 ± 1.59

Data are presented as the mean (HU) ± SD. Attenuation, SNR, and CNR are reported for LP and PV. Abbreviations: HU, Hounsfield Unit; LP, liver parenchyma; PV, portal vein; CNR, contrast-to-noise ratio; SNR, signal-to-noise ratio.

**Table 5:** Qualitative assessment of image quality.

	DECT at 60 keV	SECT	*p* Value
Image overall quality (range)	3.95 (3–5) AC = 0.769	3.83 ± 0.7 (2–5) AC = 0.54	0.287
Contrast enhancement (range)	4.08 (3–5) AC = 0.83	3.35 ± 0.7 (2–5) AC = 0.23	<0.001
Image noise (range)	4.55 ± 0.6 (3–5) AC = 0.83	3.58 ± 0.8 (2–5) AC = 0.40	<0.001

Note: data are presented as mean ± standard deviation. Abbreviations: SECT, single-energy computed tomography; DECT, dual-energy computed tomography, AC; agreement coefficient.

**Table 6:** Quantitative assessment of image quality.

	DECT at 60 keV	SECT	*p* Value
aHU_LP_	119.30 ± 11.1	109.8 ± 9.0	<0.001
CNR_LP_	6.59 ± 1.5	4.04 ± 1.5	<0.001
SNR_LP_	12.95 ± 1.8	7.64 ± 1.7	<0.001
HU_PV_	200.01 ± 37.0	149.6 ± 16.6	<0.001
CNR_PV_	14.47 ± 4.3	6.9 ± 2.2	<0.001
SNR_PV_	13.44 ± 3.6	10.43 ± 2.6	<0.001
Image Noise	9.93 ± 1.7	13.98 ± 2.5	<0.001

Note: data are presented as mean ± standard deviation. Abbreviations: SECT, single-energy computed tomography; DECT, dual-energy computed tomography; LP, live; PV, portal vein; parenchyma HU, Hounsfield Unit; aHU, average Housnfield Unit (mean between left and right lobe); CNR, contrast-to-noise ratio; SNR, signal-to-noise ratio.

## Data Availability

Data are contained within the article.

## Abbreviations

The following abbreviations are used in this manuscript:

BMI	Body Mass Index
CT	Computed Tomography
DECT	Dual-energy Computed Tomography
keV	Kiloelectronvolts
kV	Kilovolts
ICM	Iodinated Contrast Medium
PV	Portal Vein
SD	Standard Deviation
SECT	Single-energy Computed Tomography
VMI	Virtual Monochromatic Image
